# Semiautomated pipeline for quantitative analysis of heart histopathology

**DOI:** 10.1186/s12967-023-04544-2

**Published:** 2023-09-26

**Authors:** Patrick Droste, Dickson W. L. Wong, Mathias Hohl, Saskia von Stillfried, Barbara M. Klinkhammer, Peter Boor

**Affiliations:** 1 LaBooratory of Nephropathology, Institute of Pathology, Medical Faculty, RWTH Aachen University, Aachen, Germany; 2 Division of Nephrology and Clinical Immunology, Medical Faculty, RWTH Aachen University, Aachen, Germany; 3 Department of Internal Medicine III, University Hospital, Saarland University, Homburg, Germany

**Keywords:** ImageJ, Heart histology, Heart histopathology, Heart disease, Hypertrophy of cardiomyocytes

## Abstract

**Background:**

Heart diseases are among the leading causes of death worldwide, many of which lead to pathological cardiomyocyte hypertrophy and capillary rarefaction in both patients and animal models, the quantification of which is both technically challenging and highly time-consuming. Here we developed a semiautomated pipeline for quantification of the size of cardiomyocytes and capillary density in cardiac histology, termed HeartJ, by generating macros in ImageJ, a broadly used, open-source, Java-based software.

**Methods:**

We have used modified Gomori silver staining, which is easy to perform and digitize in high throughput, or Fluorescein-labeled lectin staining. The latter can be easily combined with other stainings, allowing additional quantitative analysis on the same section, e.g., the size of cardiomyocyte nuclei, capillary density, or single-cardiomyocyte protein expression. We validated the pipeline in a mouse model of cardiac hypertrophy induced by transverse aortic constriction, and in autopsy samples of patients with and without aortic stenosis.

**Results:**

In both animals and humans, HeartJ-based histology quantification revealed significant hypertrophy of cardiomyocytes reflecting other parameters of hypertrophy and rarefaction of microvasculature and enabling the analysis of protein expression in individual cardiomyocytes. The analysis also revealed that murine and human cardiomyocytes had similar diameters in health and extent of hypertrophy in disease confirming the translatability of our murine cardiac hypertrophy model. HeartJ enables a rapid analysis that would not be feasible by manual methods. The pipeline has little hardware requirements and is freely available.

**Conclusions:**

In summary, our analysis pipeline can facilitate effective and objective quantitative histological analyses in preclinical and clinical heart samples.

**Supplementary Information:**

The online version contains supplementary material available at 10.1186/s12967-023-04544-2.

## Introduction

Diseases affecting the heart are a major burden in Western populations. Preclinical and clinical research plays an important role in better understanding and treating heart diseases [1]. The majority of these diseases are associated with cellular morphological changes, which can be evaluated using histology. In both animal models and patients, histopathology analyses of the heart (or myocardial biopsies) remain of high relevance. Some of the commonly analyzed histological changes include hypertrophy of cardiomyocytes, associated with enlargement of the cardiomyocyte nuclei, changes in the number of capillaries, particularly microvascular rarefaction, infiltration by inflammatory cells, and interstitial fibrosis. Interstitial fibrosis is relatively easy to quantify with histochemical stainings, e.g., Sirius red, Masson trichrome, or immunohistochemistry. These can be evaluated by available software solutions, e.g., as a proportion of positively stained area [2, 3]. Infiltration by inflammatory cells can be best evaluated using immunohistochemistry (or immunofluorescence) using specific markers for inflammatory cells, and various quantification approaches exist for this as well. Fibrosis and inflammation are often assessed, in part due to the relative ease to analyze them. Hypertrophy of cardiomyocytes, enlargement of cardiomyocyte nuclei, and capillary rarefaction are not as easy to analyze on histology. This is particularly challenging when analyzing the relationship between morphometric features, e.g., the ratio of cardiomyocytes to capillaries. Standardized, robust, and simple methods are largely missing [4]. Manual evaluation of these parameters is still commonly done but is very time-consuming, prone to interobserver variability, and detailed descriptions of the methods are not seldom missing [5–10]. Here we developed a histopathology evaluation package of pipelines to facilitate histological analysis of cardiomyocyte size, cardiomyocyte nuclei size, capillary density, and single cardiomyocyte protein or RNA expression evaluation, which we called HeartJ. HeartJ uses images of histochemical or immunofluorescence staining. The use of histochemical staining enables the use of bright-field whole slide scanners, which facilitates high-throughput workflow and reduces effort and costs. Using HeartJ does not require any special hardware or knowledge of computer science.

## Materials and methods

### Mice

The animal study was performed following German law for the protection of animals. The investigation conforms to the guide for the Care and Use of Laboratory Animals published by the United States National Institutes of Health (Eighth edition; revised 2011). The study was approved by the regional Animal Welfare Inspectorate (Saarländisches Landesamt für Verbraucherschutz No21/2014). Transverse-aortic constriction (TAC) was performed in five weeks old C57BL/6N mice (Charles River, Germany). C57/BL/6N mice were anesthetized with a 1:10 dilution of 100 mg/kg ketamine and 10 mg/kg xylazine and connected to a volume-cycled rodent ventilator (Harvard Apparatus) using a 20 gauge catheter. Constriction of the aorta was performed by tying a 7–0 nylon suture ligature against a 27 gauge needle yielding a transverse aortic constriction of 65–70%. Control mice underwent a sham operation in which the nylon suture was placed, but not pulled tied. After 6 weeks, animals were euthanized by injecting an overdose of a mixture of ketamine hydrochloride (100 mg/kg body weight) and xylazine hydrochloride (10 mg/kg body weight). Hearts were rapidly excised and processed for further experiments.

### Human samples

The human specimens were selected from autopsies performed at RWTH Aachen University in a period from 2008 to 2019. Seven cases with Aortic (valve) stenosis (ICD-10 code I35.0) and 14 cases with no clinical or autopsy findings of cardiac disease were selected. For all autopsies, legal authorization was obtained from the next of kin of the deceased person. There were no ethical or professional concerns regarding the study raised by the local ethics committee 042/17. Several formalin-fixed paraffin-embedded blocks were available for each heart. We selected one from the left ventricle for analysis that did not show focal pathologies, such as infarction or scar formation.

### Histochemistry and immunofluorescence staining

All formalin-fixed specimens were dehydrated, paraffin-embedded, and cut into 4 µm thin sections. A modified Gomori silver staining for reticulum was performed with pretreatment by thiosemicarbazide and periodic acid, followed by methenamine silver solution and further processing to silvering by gold chloride and nitric acid formalin. A counterstain was performed with hematoxylin and eosin.

For immunofluorescence staining, slides were rehydrated and heat-induced antigen retrieval was performed in a citrate buffer. Slides were incubated with fluorescein-coupled WGA (wheat germ agglutinin; 1:100; FL-1021; Vector Laboratories; Burlingame; CA; USA; RRID:AB_2336866) and CD31 (cluster-of-differentiation 31; 1:50; AF3628; R&D Systems; Minneapolis; USA; RRID:AB_2161028) or pro-ANP (pro Atrial natriuretic peptide; 1:100; ab91250; Abcam; Cambridge; UK; RRID:AB_2049188). Anti-goat (1:200; BA-5000; Vector Laboratories, Burlingame; CA; USA; RRID:AB_2336126) or anti-rabbit secondary antibodies (1:200; BA-1000; Vector Laboratories; Burlingame; CA; USA; RRID:AB_2313606) were used, respectively, and VECTASTAIN^®^ Elite ABC-HRP Kit (Vector Laboratories; Burlingame; CA; USA) was used for amplification. Staining was developed with Opal^™^ 570 fluorophores (PerkinElmer Life and Analytical Sciences; Boston; MA; USA), and nuclei were counterstained with DAPI.

### Real-time quantitative PCR

Gene expression analysis was performed by real-time quantitative PCR (RT qPCR). Total RNA was extracted from the left ventricular tissue of the mouse using peqGold TriFast (30–2010; VWR; Darmstadt, Germany) extraction reagent per manufacturer´s protocol. Genomic DNA impurities were removed by DNase treatment (13–1091; VWR; Darmstadt, Germany), and cDNA was synthesized by reverse transcription using the HighCap cDNA RT Kit (4368814; Applied Biosystems; Waltham, MA, USA) according to the manufacturer´s protocol. RT qPCR was conducted in a StepOne plus thermocycler (Applied Biosystems; Waltham, MA, USA) using TaqMan GenEx Mastermix (4369016, Applied Biosystems; Waltham, MA, USA). Signals were normalized to corresponding glyceraldehyde-3-phosphate dehydrogenase (GAPDH) controls. No template controls were used to monitor for contaminating amplifications. The ΔCt was used for statistical analysis and 2^−ΔΔCt^ for data presentation. Probes used to amplify the transcripts were as follows (purchased by Applied Biosystems; Waltham, MA, USA): mouse GAPDH (Mm99999915_g1), mouse atrial natriuretic peptide (ANP) (Mm01255747_g1), mouse brain natriuretic peptide (BNP) (Mm01255770_g1), mouse CTGF (Connective tissue growth factor) (Mm01192932_g1).

### HeartJ Pipeline

**Fig. 1:**
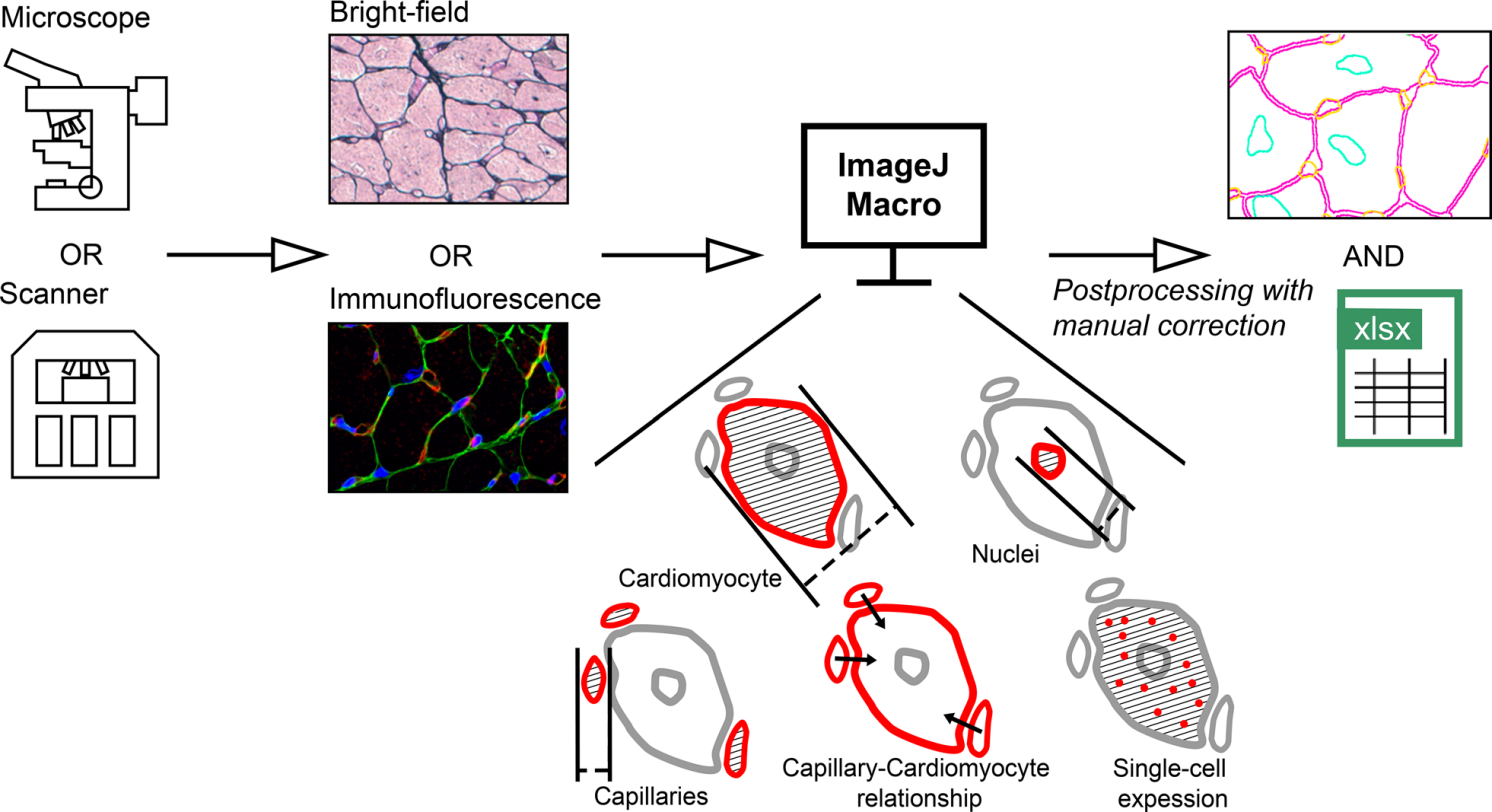
Overview of the analysis pipeline HeartJ. Gomori silver staining (Bright-field) or immunofluorescence staining (WGA (green), DAPI (blue), CD31 (red)) images can be acquired by microscopes or scanners. Macros created for different types of images can analyze the size of cardiomyocytes, nuclei of cardiomyocytes, and capillaries. In addition, the relationship between capillaries and cardiomyocytes can be analyzed, as well as the expression of an intracardiomyocytic signal. The results are exported as an Excel file, and an overview of the analyzed image is also created, enabling manual correction of the results

In the general workflow of HeartJ, the first step is image acquisition (Fig. 1). Since the macro uses image files, images can be acquired from a microscope or whole slide scanners. Supported file formats are all standard image formats like BMP, GIF, JPEG, PNG, or TIFF. We have developed various macros for different combinations of parameters, these are described in detail below. All macros generate an Excel file and an overlay image with the visualized results, which allows postprocessing by manual corrections as well as further individual quality control and evaluations. All macros with instructions are freely available on GitHub (https://github.com/PaDroste/HeartJ).

### Image acquisition

The slides with Gomori silver staining from mice and humans were digitized using Aperio AT2 whole slide scanner (Leica Biosystems, Wetzlar, Germany) with a 40 × objective. On the whole slide image (WSI) of each mouse, three regions (433 × 270 µm) were selected respectively from the inner wall of the left ventricle with cross sections of cardiomyocytes, the mid-wall of the left ventricle with longitudinal sections of cardiomyocytes, the outer wall of the left ventricle with cross sections of cardiomyocytes, and the inner wall of the right ventricle with cross sections of cardiomyocytes (Additional file 1: Fig. S1). Since significantly more tissue is present on slides of human hearts, five larger regions were selected for analysis from human hearts, i.e., 865 × 476 µm. The selected regions were exported as image files in JPEG format. Pictures from immunofluorescence stainings with a size of 332 × 220 µm were taken from the inner wall of the left ventricle with cross sections of cardiomyocytes using Zeiss Axio Imager 2 with a 40 × objective and ApoTome 2 (Carl Zeiss AG, Oberkochen, Germany).

### ImageJ

We have used the latest available version of ImageJ, 1.54a.

### Processing of gomori silver staining

**Fig. 2:**
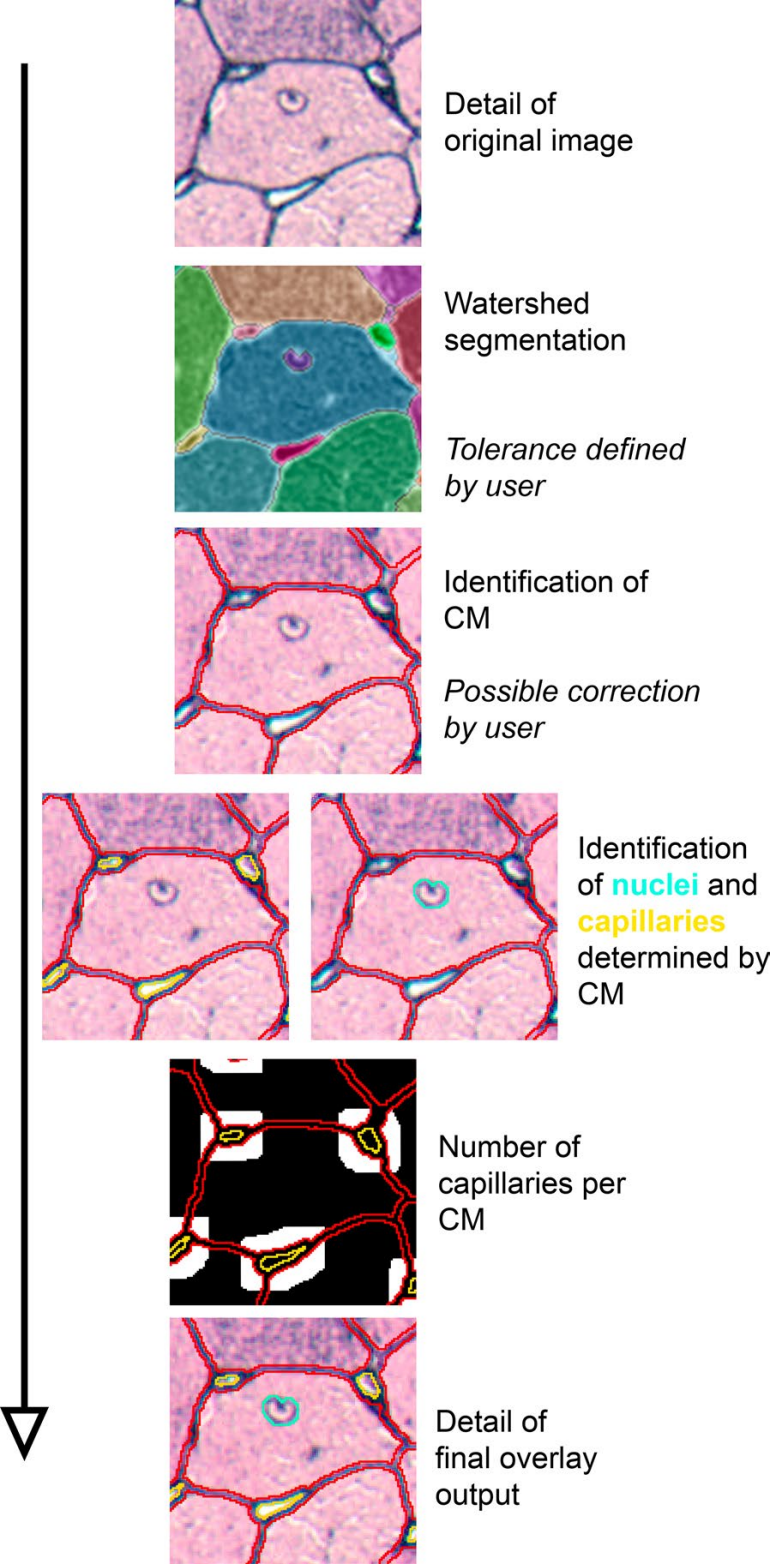
Processing of Gomori silver staining in ImageJ. First, segmentation is performed using watershed segmentation. The user defines the tolerance and can add missing lines if necessary. Cardiomyocytes (CM) are identified by size and color in all instances. Capillaries are identified by their position between cardiomyocytes and size. Nuclei of cardiomyocytes are identified by size and location within cardiomyocytes. The ratio of cardiomyocytes to capillaries is evaluated. An overview image with all evaluated instances is created

The macro for processing Gomori silver staining bright-field images was coded in the ImageJ macro language. The images of Gomori silver staining were first converted to black and white images. The analysis was based on watershed segmentation (Fig. 2). This was done by the ImageJ plugin MorphoLibJ [11]. To achieve high flexibility and quality of the analysis, we decided that the tolerance of the watershed segmentation should be defined by the user. Especially in case of poorer quality of the staining, this has proven to be advantageous compared to faster-automated analysis. There was also an option to add manually any missing lines in the segmentation. In the next step, the instances were sorted, and they were assigned to cardiomyocytes, nuclei of cardiomyocytes, or capillaries. Instances classified as cardiomyocytes were identified by staining and size. In the Gomori silver staining, the muscle tissue is reddish. Based on 40 stainings from our laboratory, we have defined the appropriate color range (HSB: 240–360°; 32–100%; 54–100%). As another factor, the minimum size was used. For this, we have selected a minimum of 60 µm^2^ from over 50,000 cardiomyocytes from healthy and hypertrophic murine hearts.

For the human hearts, we used a minimum of 50 µm^2^, after evaluating over 80,000 cardiomyocytes. Cardiomyocytes that were at the edge of the image and captured partially were excluded. Cardiomyocyte nuclei were defined as instances within cardiomyocytes. The minimum and maximum sizes were also defined from over 6000 nuclei as 6 µm^2^ to 100 µm^2^. Capillaries were defined as instances outside the cardiomyocytes. Since these were not stained and thus could easily be mistaken for artefacts, a minimum and maximum size were also defined from over 40,000 capillaries as 1.5 µm^2^ to 100 µm^2^. All limits were rounded up or down very roughly to be sure not to miss any instance. Instances that did not belong to one of the three structures were removed. Next, the relationship between cardiomyocytes and capillaries was analyzed by measuring the number of capillaries bordering a cardiomyocyte. Only capillaries directly bordering the cardiomyocytes are counted, therefore a maximum distance of 2 µm was defined. All results were exported from ImageJ by the plugin “Read and Write Excel (v1.1.3)”. An overlay image and color-coded instances were created. The Excel file contained a summary and detailed data for each instance. This included the area, Feret diameters, and number of capillaries per cardiomyocyte. Postprocessing with subsequent editing was possible from the result report, e.g., a manual correction to exclude incorrectly segmented structures. All manipulations of control steps performed in this paper have been done in a blinded manner and performed by a trained histopathology expert. For the analysis of the size of cardiomyocytes, we preferred to use the minimum Feret diameter (MinFeret). This is the minimum distance of two parallel lines restricting an object. The MinFeret parameter is independent of the cutting angle of the cardiomyocytes in contrast to the area.

### Processing of WGA+CD31+DAPI

**Fig. 3:**
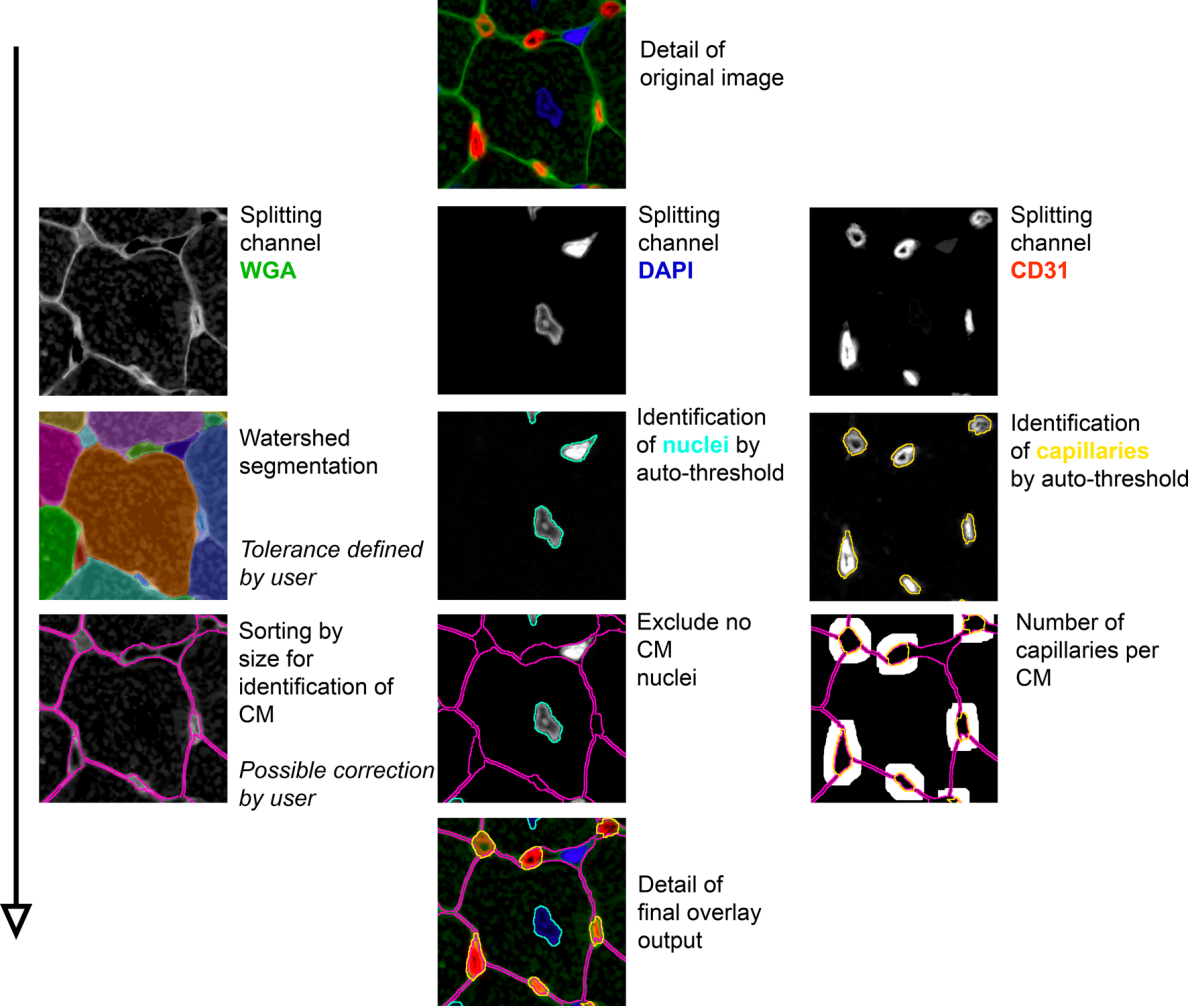
Processing of WGA+CD31+DAPI in ImageJ. Merge images are split into single channels first. The WGA channel is segmented using watershed segmentation. The user defines the tolerance and can add missing lines if necessary. The cardiomyocytes (CM) are identified by their size. Nuclei of cardiomyocytes and capillaries are identified in their appropriate channel by auto-threshold. By using the instances of the cardiomyocytes noncardiomyocyte nuclei can be excluded and the relationship of capillaries per cardiomyocyte can be evaluated. An overview image with all evaluated instances is created

The macro for processing of triple stainings of WGA +CD31+DAPI operated similarly to the macro for Gomori silver staining (Fig. 3). First, the three different channels were split. The channel with WGA staining was segmented using a watershed segmentation [11]. Compared to the macro for Gomori silver staining, no identification of cardiomyocytes by staining was possible, so cardiomyocytes were identified by size only. The nuclei were identified by an auto-threshold. This was possible due to the high contrast of the DAPI staining. Based on the cardiomyocytes, nuclei that were located outside of cardiomyocytes were excluded to measure only the nuclei of the cardiomyocytes. The capillaries were also identified by an auto-threshold after excluding the area belonging to cardiomyocytes. The analysis of capillaries bordering a cardiomyocyte and all further steps were performed in the same way as described for macro for Gomori silver staining.

### Processing of WGA+DAPI+intracardiomyocytic-expression (e.g., pro-ANP)

Compared to the macro for WGA+DAPI+CD31, this macro analyzes an intracardiomyocytic signal instead of CD31, e.g., pro-ANP. The macro proceeds similarly to the macro for WGA+CD31+DAPI. Instead of analyzing the channel with CD31, the intracardiomyocytic signal was identified by an auto-threshold, and the percentage of positive area per cardiomyocyte and cardiomyocyte nuclei was reported as a result (Additional file 1: Fig. S2).

### Data analysis

GraphPad Prism 9 was used for data analysis. F test was used to check for similar variances and the Shapiro–Wilk test was used to check for normal distribution. Unless otherwise indicated, the data were parametric and all values were expressed as means ± standard deviation. If the data were non-parametric, this was indicated by specifying the median and 25th and 75th centiles. Student`s unpaired t-test was applied to check for differences between the two groups for parametric data. Mann–Whitney-U-Test was applied to check for differences between two groups for nonparametric data. If not specially marked, data in the graphs were presented as individual values (points) per animal or human, mean values and standard deviations (error bars) per group with p-value. For the correlation matrix, Spearman correlation was used. Statistical significance was defined as p < 0.05. Figures were created with Adobe Photoshop 2023.

## Results

### Basic characterization of the murine hearts from TAC experiment

In C57/BL6N mice with TAC and Sham, the heart weight and ratio of heart to body weight were significantly increased in TAC mice (Additional file 1: Fig. S3A). RT qPCR showed a significant upregulation in TAC mice of ANP and BNP which are associated with heart failure and cardiac hypertrophy (Additional file 1: Fig. S3B) [12]. CTGF was also significantly upregulated, which is associated with hypoxia and ischemia [13].

### Basic characterization of the human cohort

The human cohort included a total of 21 autopsy cases. Seven cases had a record of a diagnosis of ICD-10 code I35.0, aortic valve stenosis, and autopsy-confirmed diagnosis of cardiac hypertrophy with a mean age of 75 years, and 14 cases had no clinical or autopsy findings of cardiac disease with a mean age of 34 years. Accordingly, patients with aortic valve stenosis showed significantly higher heart weight and left myocardial thickness compared to patients without aortic valve stenosis (Additional file 1: Fig. S4).

### HeartJ analysis of the gomori silver staining

**Fig. 4:**
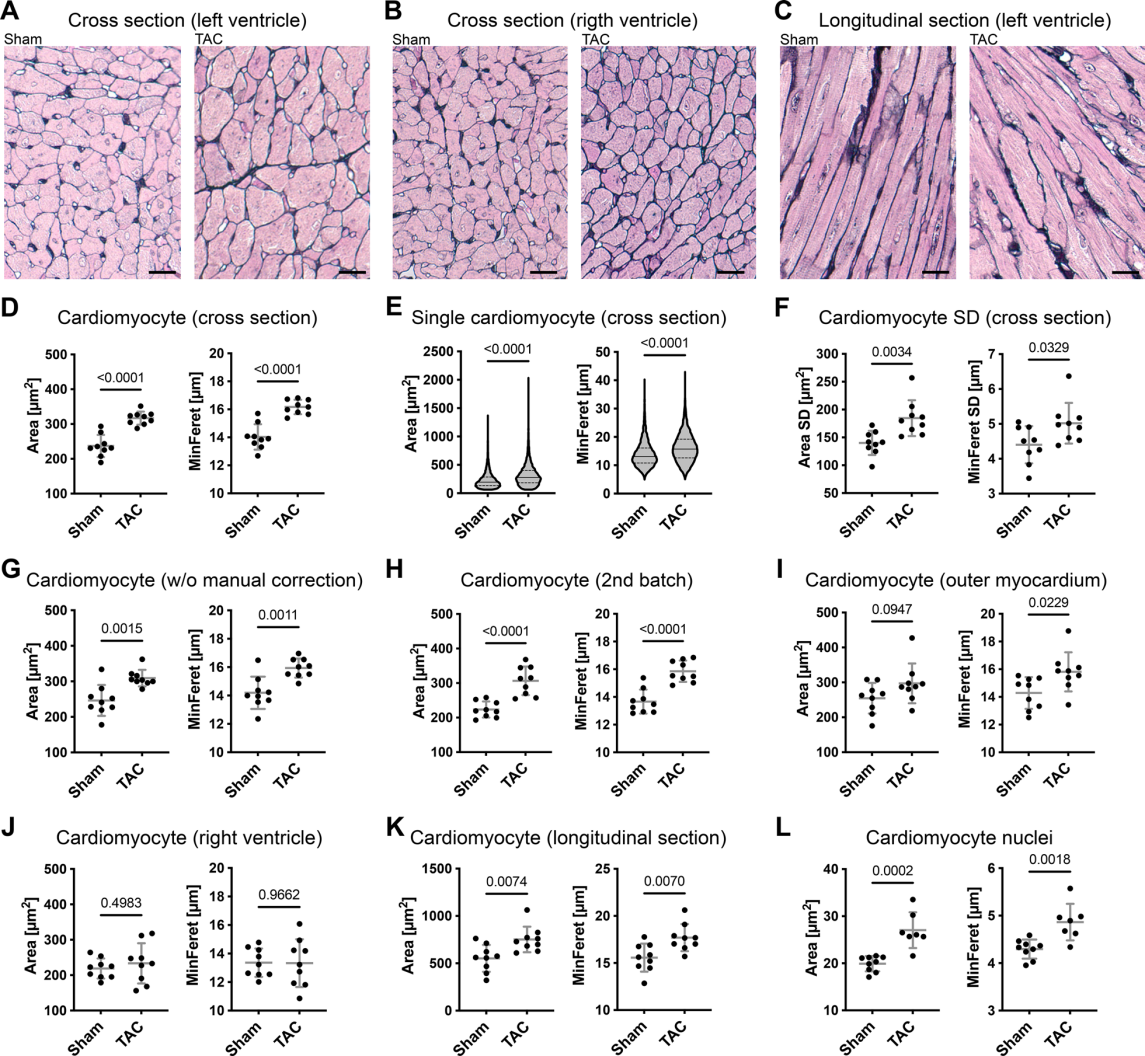
Evaluation of TAC experiment with Gomori silver staining. **A** Representative images of cross sections from the inner wall of the left ventricle, **B** of cross sections from the inner wall of the right ventricle and **C** of longitudinal sections from the mid-wall of the left ventricle of sham and TAC mice, Gomori silver staining (scale = 20 µm). **D** Cardiomyocyte area and MinFeret of sham mice (n = 9) and TAC mice (n = 9) from three cross section images per animal of the inner wall of the left ventricle with manual correction as mean value per animal, **E** as violin diagram of single cardiomyocytes of sham mice (n = 7401) and TAC mice (n = 6069), Median (horizontal line), 25th and 75th centiles (dotted line), and **F** Standard deviation (SD) of single cardiomyocytes per animal. **G** Cardiomyocyte area and MinFeret of sham mice (n = 9) and TAC mice (n = 9) from three cross section images per animal of the inner wall of the left ventricle without manual correction, **H** from three cross section images per animal of the inner wall of the left ventricle from the second batch with manual correction and **I** from three cross section images per animal of the outer wall of the left ventricle with manual correction and **J** the inner wall of the right ventricle and with manual correction. **K** Cardiomyocyte area and MinFeret of sham mice (n = 9) and TAC mice (n = 9) from three longitudinal section images per animal of the mid-wall of the left ventricle with manual correction. **L** Cardiomyocyte nuclei area and MinFeret of sham mice (n = 9) and TAC mice (n = 9) from three cross section images per animal of the inner wall of the left ventricle with manual correction

The standard staining for histopathology analysis of cardiac tissue is hematoxylin and eosin, but this staining does not allow reliable identification of individual cardiomyocytes (Additional file 1: Fig. S5). Therefore, we have used the Gomori silver staining which easily allows the distinction between cardiomyocytes (Fig. 4A, B, C), and developed HeartJ for its analysis. In the TAC model, we used three images from the inner region of the left ventricle on which the cardiomyocytes were visible in each cross section (Additional file 1: Fig. S1). Compared to sham mice, cardiomyocytes of TAC mice had significantly increased cardiomyocyte area by 34% (237 ± 32 µm^2^ vs 317 ± 19 µm^2^; p < 0.0001) and increased minimum Feret Diameter (MinFeret) by 14% (14 ± 0.9 µm vs 16 ± 0.5 µm; p < 0.0001; Fig. 4D). When cardiomyocyte values were considered individually and plotted as a violin diagram, there was also a significant difference between sham mice and TAC mice in the area (median 194 µm^2^ (25th centile 134 µm^2^; 75th centile 286 µm^2^) vs median 279 µm^2^ (25th centile 185 µm^2^; 75th centile 403 µm^2^); p < 0.0001) and MinFeret (median 13 µm (25th centile 11 µm; 75th centile 16 µm) vs median 16 µm (25th centile 13 µm; 75th centile 19 µm); p < 0.0001; Fig. 4E) with a higher distribution in the TAC mice. Comparing the standard deviation of the cardiomyocytes of the individual animals, there is a significant difference for the area (140 ± 22 µm^2^ vs 185 ± 32 µm^2^; p = 0.0034) and MinFeret (4 ± 0.5 µm vs 5 ± 0.5 µm; p = 0.0329; Fig. 4F), this indicated significant higher intraindividual variability of cardiomyocytes in the TAC mice. Approximately, 750 cardiomyocytes were analyzed per animal. This analysis was manually corrected, so a total of 13% of false cardiomyocytes were excluded from the analysis. Even without the manual correction, the results were still significant for the area (246 ± 43 µm^2^ vs 309 ± 23 µm^2^; p = 0.0015) and MinFeret (14 ± 1.1 µm vs 16 ± 0.7 µm; p = 0.0011; Fig. 4G). The selection of three areas of the inner left ventricle could be limited by a selection bias. To exclude this, we selected three different areas of the inner region of the left ventricle and evaluated them with manual correction. The result showed similar significant results and absolute values for the area (223 ± 23 µm^2^ vs 306 ± 41 µm^2^; p < 0.0001) and MinFeret (14 ± 0.9 µm vs 16 ± 0.8 µm; p < 0.0001; Fig. 4H). This suggested that three selected areas with approximately 750 cardiomyocytes are representative of this disease model. The MinFeret provided more consistent results since it is independent of the plane in which individual cardiomyocytes are cut. We also evaluated three regions of the outer left ventricle. The result showed no significant difference between sham mice and TAC mice for the area of cardiomyocytes (254 ± 44 µm^2^ vs 297 ± 57 µm^2^; p = 0.0947), but a significant difference in the MinFeret of cardiomyocytes (14 ± 1.1 µm vs 16 ± 1.4 µm; p = 0.0229), although with a lower significance compared to the inner of the left ventricle (Fig. 4I).

In three regions from the right ventricle, we detected smaller cardiomyocytes with no significant differences in the area (219 ± 28 µm^2^ vs 233 ± 59 µm^2^; p = 0.4983) and MinFeret (13 ± 1.0 µm vs 13 ± 1.7 µm; p = 0.9662) between sham mice and TAC mice (Fig. 4J). We tested the macro on images with longitudinally aligned cardiomyocytes from the mid-wall of the left ventricle. While we still observed significant differences between the groups regarding area (552 ± 142 µm^2^ vs 752 ± 135 µm^2^; p = 0.0074) and MinFeret (16 ± 1.5 µm vs 18 ± 1.5 µm; p = 0.0070), the data showed an overall higher cell size and more variability, with a lower level of significance (Fig. 4K). Due to the variability of Gomori silver staining, it is challenging to specifically measure capillary density in all animals (Additional file 1: Fig. S6). The lack of sufficient segmentation of capillaries resulted in an incorrect low number of capillary contacts. Also, not all nuclei could be segmented in this staining, albeit a statistically significant increase in the area (20 ± 1.6 µm^2^ vs 27 ± 3.8 µm^2^; p = 0.0002) and MinFeret (4.3 ± 0.2 µm vs 4.9 ± 0.4 µm; p = 0.0018) of the nuclei of the cardiomyocytes of the inner region of the left ventricle could be observed (Fig. 4L).

The whole HeartJ pipeline procedure took less than one minute to analyze an image without manual corrections. The time required for a manual correction of one image depended on the image quality and was between 2 and 6 min. Compared to completely manual segmentation and analysis, which was not feasible for 750 cardiomyocytes per sample with both approaches, but particularly without manual correction, strongly reduced the required time.

**Fig. 5:**
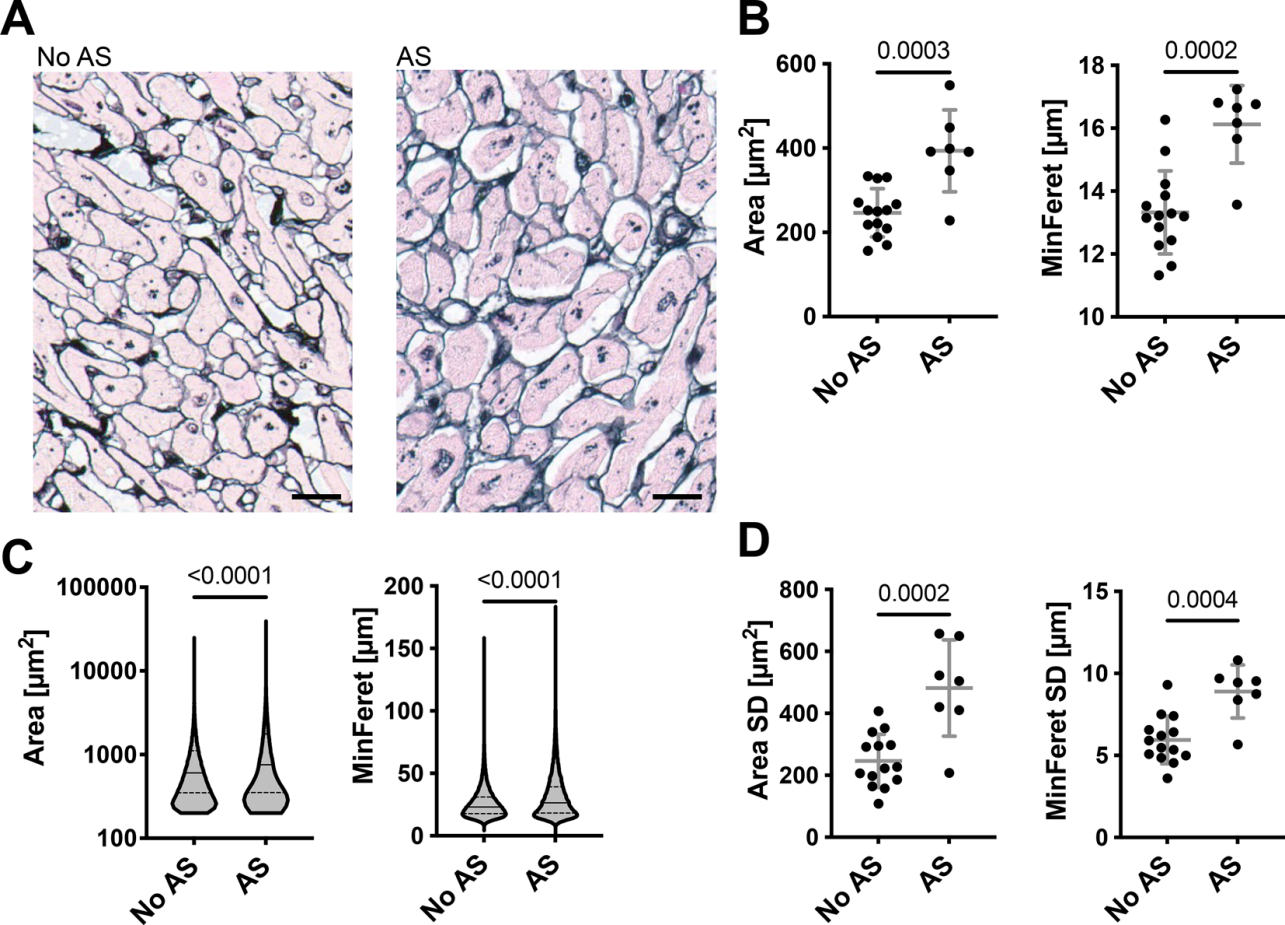
Evaluation of human cohort with Gomori silver staining. **A** Representative images of cross sections of no aortic stenosis (AS) and AS (scale = 20 µm). **B** Cardiomyocyte area and MinFeret of no AS (n = 14) and AS (n = 7) from five cross section images per case without manual correction **C** and as violin diagram of single cardiomyocytes of no AS (n = 60 733) and AS (n = 21 644). Median (horizontal line), 25th and 75th centiles (dotted line). **D** Standard deviation (SD) of cardiomyocyte area and MinFeret of no AS (n = 14) and AS (n = 7) from five cross section images per patient without manual correction

We next analyzed the Gomori silver staining of the autopsy cohort of hearts with and without aortic valve stenosis using the same HeartJ pipeline. We selected five areas with cross sectioned cardiomyocytes from each patient's slide. Because of the high number of cardiomyocytes (approximately 4000 per case), and the reassuring results from the TAC experiment, we omitted manual correction. Compared to cases without aortic valve stenosis, hearts of patients with stenosis had a significant increase in the cardiomyocyte area by 60% (246 ± 57 µm^2^ vs 394 ± 97 µm^2^; p = 0.0003) and MinFeret by 23% (13 ± 1.3 µm vs 16 ± 1.3 µm; p = 0.0002; Fig. 5A, B). Comparison of the individual cardiomyocytes also revealed a significant difference between area (median 604 µm^2^ (25th centile 349 µm^2^; 75th centile 1113 µm^2^) vs median 756 µm^2^ (25th centile 352 µm^2^; 75th centile 1769 µm^2^); p < 0.0001) and MinFeret (median 23 µm (25th centile 18 µm; 75th centile 31 µm) vs median 26 µm (25th centile 18 µm; 75th centile 39 µm); p < 0.0001; Fig. 5C). There is a significantly higher intraindividual variability of cardiomyocytes in the cases with aortic valve stenosis, indicated by a significantly increased standard deviation of area (246 ± 86 µm^2^ vs 482 ± 155 µm^2^; p = 0.0002) and MinFeret (6 ± 1.5 µm vs 9 ± 1.6 µm; p = 0.0004) of individual cardiomyocytes per case (Fig. 5D). The MinFeret of human cardiomyocytes was very similar to that of murine cardiomyocytes, but the variability of MinFeret in the individual cardiomyocytes was greater in the human cohort.

### Capillary density and pro-ANP Expression analysis

**Fig. 6:**
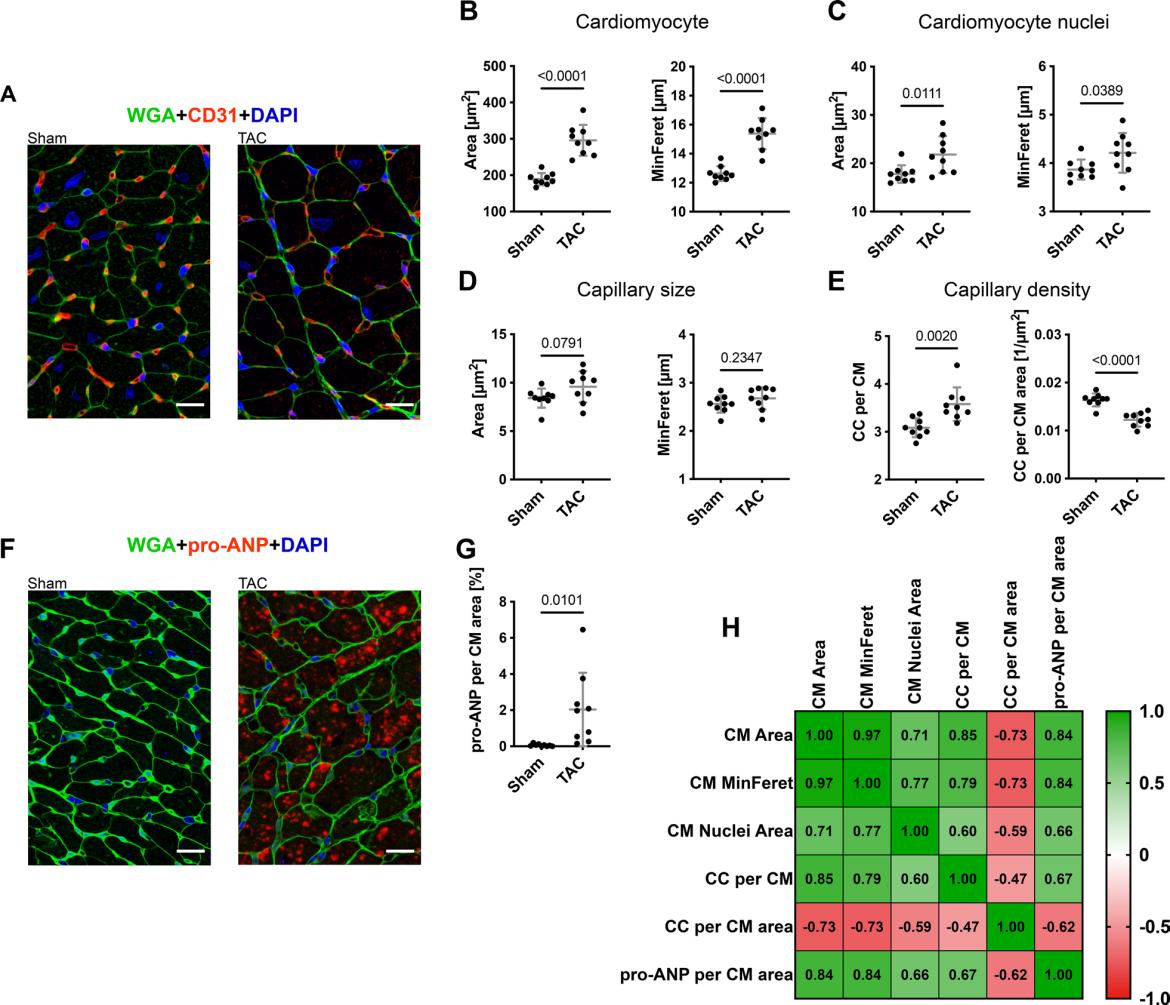
TAC experiment capillary density and pro-ANP expression. **A** Representative images of cross sections of sham and TAC mice, WGA (green), CD31 (red), and DAPI (blue) (scale = 20 µm). **B** Cardiomyocyte area and MinFeret of sham mice (n = 9) and TAC mice (n = 9) from three cross section images per animal of the inner wall of the left ventricle with manual correction **C** and their nuclei area and MinFeret with manual correction. **D** Capillary area and MinFeret of sham mice (n = 9) and TAC mice (n = 9) from three cross section images per animal of the inner wall of the left ventricle with manual correction. **E** Capillary contacts (CC) of one cardiomyocyte and ratio from CC to the area of the cardiomyocyte (CM). **F** Representative images of cross sections of sham and TAC mice, WGA (green), pro-ANP (red), and DAPI (blue). (scale = 20 µm). **G** Pro-ANP expression of sham mice (n = 9) and TAC mice (n = 9) per area of cardiomyocyte (CM) in percent (%) from three cross section images per animal of the inner wall of the left ventricle with manual correction. **H** Spearman correlation matrix of cardiomyocyte (CM) area, cardiomyocyte MinFeret, cardiomyocyte nuclei area, capillary contacts, Ratio of capillary contacts (CC) and cardiomyocyte area, and pro-ANP expression in percent of cardiomyocyte area

Because capillary density could not be reliably analyzed in the Gomori silver staining, we developed HeartJ also for immunofluorescence stainings of the murine cohort using WGA, CD31, and DAPI (Fig. 6A). As in the evaluation with Gomori silver staining, a significant increase in the area of the cardiomyocytes (189 ± 17 µm^2^ vs 296 ± 43 µm^2^; p < 0.0001) and the MinFeret of the cardiomyocytes (13 ± 0.5 µm vs 15 ± 1.1 µm; p < 0.0001) was observed between sham mice and TAC mice in the inner region of the left ventricle. (Fig. 6B). Using DAPI, from the approximately 530 cardiomyocytes per mouse in this analysis, 170 nuclei could be detected and evaluated. This is not unexpected in a cross section of the heart due to the large length and volume of cardiomyocytes compared to the nucleus. Compared to sham controls, TAC mice had a significant increase in the nuclei area (18 ± 1.8 µm^2^ vs 22 ± 3.8 µm^2^; p = 0.0111) and MinFeret (3.9 ± 0.2 µm vs 4.2 ± 0.4 µm; p = 0.0389; Fig. 6C). The size of the capillaries showed no significant difference between the groups (Fig. 6D). Per animal, the evaluation covered approximately 900 capillaries. In sham animals, one cardiomyocyte had contact with three capillaries whereas in TAC animals this number was higher (3.1 ± 0.2 vs 3.6 ± 0.4; p = 0.0020; Fig. 6E). This increase in the capillary contacts per cardiomyocyte may be caused by a solitary increase in the sizes of the cardiomyocytes or an increase in the number of capillaries. When normalizing the capillary contacts with the size of cardiomyocytes, the results showed a reduction of capillary contacts per area of cardiomyocytes (CC/CM Area, 0.016 ± 0.0013 1/µm^2^ vs 0.012 ± 0.0014 1/µm^2^; p < 0.0001) in sham mice compared to TAC mice (Fig. 6E).

We also developed HeartJ for expression analysis within single cardiomyocytes. We evaluated this approach by analyzing the protein expression of pro-ANP in cardiomyocytes using co-staining of WGA and pro-ANP (Fig. 6F). HeartJ enabled the analysis of pro-ANP expression on a single cardiomyocyte level. We found almost no pro-ANP expression per cardiomyocyte in the sham animals and a significant, 33-fold increase of mean single cardiomyocyte expression of pro-ANP in TAC animals (0.06% ± 0.06% vs. 2.0% ± 2.0%; p = 0.0101; Fig. 6F).

Correlation of the results of the individual animals showed a positive correlation between cardiomyocyte area, MinFeret, expression of pro-ANP, and capillary contacts. On the other hand, the rarification of microvasculature is represented by a negative correlation between these values and the capillary contacts per area of cardiomyocytes (Fig. 6H).

## Discussion

We here developed and validated a semi-automated pipeline for the quantification of cardiac histology named HeartJ. HeartJ can be applied on routine histochemical stainings, i.e. Gomori silver staining, or standard and easy-to-perform immunofluorescence stained slides, and is applicable in both patients and preclinical murine models to quantify the size and hypertrophy of cardiomyocytes. In both Gomori silver staining and immunofluorescence stainings, the size values were comparable, suggesting that both approaches provide reliable results. The Gomori silver staining is broadly used in diagnostic pathology, is quick and easy to perform, and the slides can be scanned using high-throughput whole slide scanners allowing the analysis of a large number of samples and can be archived long-term, as required in clinical pathology. It is also less prone to autofluorescence, which is usually present in human samples due to endogenous pigment and lack of tissue perfusion. Our current pipelines are not disturbed by fibrosis or inflammation, however, regions with marked destruction, e.g., within an infarction or large scars, are not suitable for this type of analysis. This also makes the pipeline applicable in clinical routine diagnostics to quantify hypertrophy of cardiomyocytes, as we showed in the human autopsy cohort with aortic stenosis with left ventricular hypertrophy. The pipeline using immunofluorescence staining allows flexible analysis of various parameters based on custom-selected staining, e.g., nuclei of cardiomyocytes, capillaries, or single-cell expression of markers in cardiomyocytes like pro-ANP.

Regarding the quantitative analysis of cardiomyocytes, we recommend using the MinFeret instead of the area, since the MinFeret is independent of the plane in which individual cardiomyocytes are cut. Technically, and because of the complex three-dimensional network of cardiomyocytes, it is not possible to cut the tissue in a way to get only cardiomyocyte cross sections. This may explain the high standard deviation of area, especially in the human cohort. The cardiomyocyte disarray that occurs in cases of cardiac hypertrophy also has a greater impact on the area than on the MinFeret [14]. We also demonstrated that in the mice with TAC, hypertrophy of the cardiomyocytes was most pronounced in the inner muscle layer of the left ventricle compared to the outer muscle layer of the left ventricle, making it the most suitable for analysis of the model. In contrast, no hypertrophy of cardiomyocytes in the right ventricle was detected, indicating that the TAC model does not affect the right ventricle at this stage.

To evaluate the capillary density in the myocardium, we recommend analyzing the capillary contacts per area of the cardiomyocytes. We observed a significant reduction in mice with TAC, consistent with a capillary rarefaction, which is in line with the RT qPCR results from CTGF reflecting decreased perfusion and hypoxia [13]. Other parameters, such as the number of capillaries per area or capillary contacts are influenced by the size of the cardiomyocytes, fibrosis or large vessels. Thereby, these parameters do not reflect that well the actual capillary density.

The structure of our pipeline has various advantages over manual analysis or already existing methods. First, using the ImageJ macro language, application-specific adaptations, and customizations are easy to implement. We have exemplarily shown this for the analysis of cardiomyocyte nuclei, pro-ANP expression in individual cardiomyocytes, and the analysis of cardiac microvasculature. Analysis of different additional immunofluorescence stainings or fluorescence in situ hybridization within the cardiomyocytes, their nuclei, or within the capillaries is thus possible. Thereby, users can further adapt HeartJ to their specific needs, e.g. redefining thresholds. The user can interfere with the macros at different steps, which allows quick quality checks, but also allows for the analysis of low-quality tissue through appropriate corrections. Second, the analysis is very cost-effective, since ImageJ is an open-source program with low hardware requirements. Third, our analysis tool spares time, particularly in combination with whole slide scanners for bright-field and immunofluorescence, and also allows using macros for batch processing. Fourth, our pipeline works on heart tissue from different mice and patients and can process bright-field and immunofluorescence slides.

Interestingly, HeartJ analysis revealed that human and murine cardiomyocytes are approximately equal in diameter and hypertrophy within a similar range. So far, there is only one publication that directly compares human and murine cardiac hypertrophy, describing different cross sectional areas of cardiomyocytes and degrees of hypertrophy, however, only quantifying cross sections of cardiomyocytes with nuclei [15]. In general, the relationship between body size and cell size is controversial and complicated by the different use of cell volume, cell cross sectional area, or cell diameter [16, 17].

Only a few publications describe approaches for the histological evaluation of cardiac muscle. One study developed an approach for the three-dimensional analysis of cardiomyocytes in rats, mice, rabbits, and sheep with thick paraffin sections using the image analysis software Imaris [18]. This approach does not apply to the broadly used two-dimensional histology analysis, higher throughput, and time-effectiveness of HeartJ. Another tool was based on Fiji and coded as a plugin, allowing the analysis of cardiomyocytes, capillaries, and fibrosis [4]. This tool is only applicable to fluorescently stained slides but has the advantage of also analyzing fibrosis in the same batch. Our pipeline can additionally determine the size of nuclei of cardiomyocytes and the single-cell expression of markers in cardiomyocytes. HeartJ is created as a macro for ImageJ, not as a plugin, so it is much easier to customize for individual needs. Another previously published tool uses ImageJ macro for the analysis of hematoxylin and eosin-stained cardiac tissues [19]. This approach measures only cardiomyocytes in transnuclear cross section, which significantly reduces the number of measured cardiomyocytes, and does not provide any other parameters. In our experience, reliable identification of individual cardiomyocytes on hematoxylin and eosin-stained cardiac tissues is not easy, or even possible.

Our pipeline has several limitations. It can only analyze parts of a WSI or individual images taken with a microscope. Although it would be possible to use WSI, these files are very large and most standard computers are not able to process such large files. Therefore, we have shown that these parts of a WSI are sufficiently representative. Our current pipeline does not include fibrosis evaluation, mainly because several straightforward approaches for fibrosis analysis already exist. However, using WGA or Gomori silver staining, the connective tissue is stained, so fibrosis can be analyzed in parallel to HeartJ [20]. Our current pipelines are not disturbed by increased fibrosis or inflammation, but regions with marked destruction of cardiac histology such as infarct scars are not suitable for this type of histological analysis. For the human cohort, we have used heart tissue from autopsies, in which, due to high autofluorescence because of erythrocytes and lipofuscin, the HeartJ immunofluorescence pipeline is not well applicable.

Our method is not based on deep learning technology, having the advantage of low hardware requirements. However, studies have shown the potential of deep learning in segmentation of histological structures in different organs and conditions [21–24]. Therefore, implementation of deep learning-based segmentation of heart histology into the pipeline could further facilitate the automation and precision of this approach. This could also enable the analysis of a broader spectrum of histological alterations, called pathomics [25].

## Conclusion

In summary, HeartJ enables the analysis of cardiac histology in various types of samples, with low hardware requirements, little software knowledge, and simple staining methods, making this method easy to implement while retaining flexibility in application scenarios. HeartJ can facilitate reproducible and high-throughput analysis potentially facilitating preclinical and clinical research. All macros with instructions are freely available on GitHub (https://github.com/PaDroste/HeartJ).

### Supplementary Information

**Additional file 1: Figure S1.** WSI of a murine heart (Gomori silver staining). Regions of the different analyses are marked: 1. Inner wall of the left ventricle with cross sections of cardiomyocytes; 2. Outer wall of the left ventricle with cross sections of cardiomyocytes 3. Mid wall of the left ventricle with longitudinal sections of cardiomyocytes 4. Inner wall of the right ventricle with cross sections of cardiomyocytes. **Figure S2.** Processing of WGA+pro-ANP+DAPI in ImageJ. Merge images are split into single channels first. The WGA channel is segmented using watershed segmentation. The user defines the tolerance and can add missing lines if necessary. The cardiomyocytes (CM) are identified by their size. Nuclei of cardiomyocytes and pro-ANP are identified in their appropriate channel by auto-threshold. By using the instances of the cardiomyocytes noncardiomyocyte nuclei can be excluded. In each instance of a cardiomyocyte, the amount of pro-ANP is evaluated. An overview image with all evaluated instances is created. **Figure S3.** Basic characterization of TAC-Experiment. A Heart weight of sham mice (n = 9) and TAC mice (n = 9) and the heart to body weight ratio. B Relative mRNA Expression of ANP, BNP and CTGF in sham and TAC mice. **Figure S4. **Heart size of human autopsy cohort. Heart weight and left myocardial thickness of autopsy cases without aortic stenosis (AS) (n = 14) and with AS (n = 7). **Figure S5.** Representative images of heart cross sections of Sham and TAC, Hematoxylin and eosin staining (scale = 20 μm). **Figure S6.** Challenges in capillary segmentation. A Representative crosssection of Gomori silver staining with many not detectable capillaries. B Representative cross-section of Gomori silver staining with detectable capillaries (scale = 20 μm).

## Data Availability

All substantial data supporting the findings of this study are included in the article and supplementary material. Additional data can be obtained from the corresponding author. The macros are freely available on GitHub (https://github.com/PaDroste/HeartJ).

## Abbreviations

ANP: Atrial natriuretic peptide
BNP: Brain natriuretic peptide
CM: Cardiomyocytes
CTGF: Connective tissue growth factor
MinFeret: Minimum Feret diameter
pro-ANP: Pro Atrial natriuretic peptide
RT qPCR: Real-time quantitative PCR
SD: Standard deviation
TAC: Transverse-aortic constriction
WGA: Wheat germ agglutinin
WSI: Whole slide image
