# Supplementary material for: Semiautomated pipeline for quantitative analysis of heart histopathology
Source: J Transl Med. 2023 Sep 26;21:666. doi: 10.1186/s12967-023-04544-2 (PMC10523682; doi:10.1186/s12967-023-04544-2)
Supplement: Supplementary file 1 — Additional file 1: Figure S1. WSI of a murine heart (Gomori silver staining). Regions of the different analyses are marked: 1. Inner wall of the left ventricle with cross sections of cardiomyocytes; 2. Outer wall of the left ventricle with cross sections of cardiomyocytes 3. Mid wall of the left ventricle with longitudinal sections of cardiomyocytes 4. Inner wall of the right ventricle with cross sections of cardiomyocytes. Figure S2. Processing of WGA+pro-ANP+DAPI in ImageJ. Merge images are split into single channels first. The WGA channel is segmented using watershed segmentation. The user defines the tolerance and can add missing lines if necessary. The cardiomyocytes (CM) are identified by their size. Nuclei of cardiomyocytes and pro-ANP are identified in their appropriate channel by auto-threshold. By using the instances of the cardiomyocytes noncardiomyocyte nuclei can be excluded. In each instance of a cardiomyocyte, the amount of pro-ANP is evaluated. An overview image with all evaluated instances is created. Figure S3. Basic characterization of TAC-Experiment. A Heart weight of sham mice (n = 9) and TAC mice (n = 9) and the heart to body weight ratio. B Relative mRNA Expression of ANP, BNP and CTGF in sham and TAC mice. Figure S4. Heart size of human autopsy cohort. Heart weight and left myocardial thickness of autopsy cases without aortic stenosis (AS) (n = 14) and with AS (n = 7). Figure S5. Representative images of heart cross sections of Sham and TAC, Hematoxylin and eosin staining (scale = 20 μm). Figure S6. Challenges in capillary segmentation. A Representative crosssection of Gomori silver staining with many not detectable capillaries. B Representative cross-section of Gomori silver staining with detectable capillaries (scale = 20 μm). [file 12967_2023_4544_MOESM1_ESM.pdf]

Semiautomated pipeline for quantitative analysis of heart histopathology

Patrick Droste[1, 2], Dickson W. L. Wong[1], Mathias Hohl[3], Saskia von Stillfried[1], Barbara M. Klinkhammer[1], Peter Boor[1,2]

[1]LaBooratory of Nephropathology, Institute of Pathology, Medical Faculty, RWTH Aachen University, Aachen, Germany

[2]Division of Nephrology and Clinical Immunology, Medical Faculty, RWTH Aachen University, Aachen, Germany

[3]Department of Internal Medicine III, University Hospital, Saarland University, Homburg, Germany

Corresponding author: Peter Boor, MD, PhD, Univ.-Prof. (pboor@ukaachen.de)

SUPPLEMENT

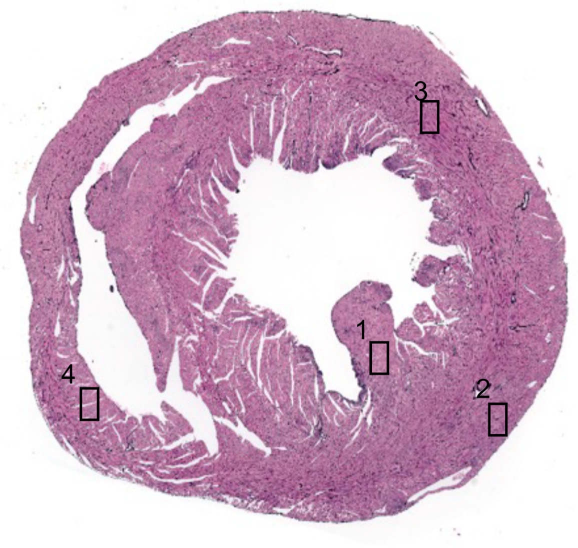

Supplement Fig 1 WSI of a murine heart (Gomori silver staining). Regions of the different analyses are marked: 1. Inner wall of the left ventricle with cross sections of cardiomyocytes; 2. Outer wall of the left ventricle with cross sections of cardiomyocytes 3. Mid wall of the left ventricle with longitudinal sections of cardiomyocytes 4. Inner wall of the right ventricle with cross sections of cardiomyocytes

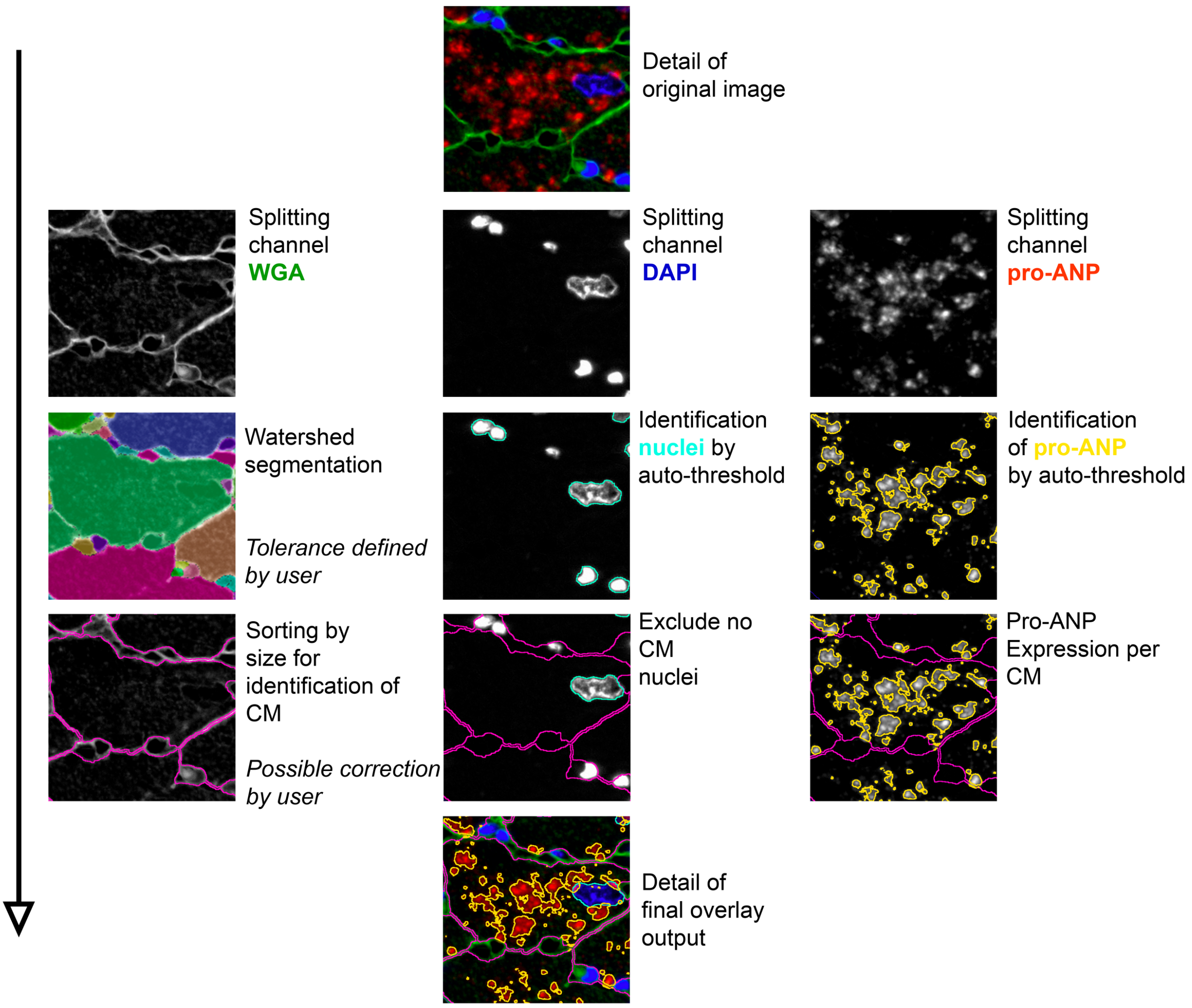

Supplement Fig 2 Processing of WGA+pro-ANP+DAPI in ImageJ. Merge images are split into single channels first. The WGA channel is segmented using watershed segmentation. The user defines the tolerance and can add missing lines if necessary. The cardiomyocytes (CM) are identified by their size. Nuclei of cardiomyocytes and pro-ANP are identified in their appropriate channel by auto-threshold. By using the instances of the cardiomyocytes noncardiomyocyte nuclei can be excluded. In each instance of a cardiomyocyte, the amount of pro-ANP is evaluated. An overview image with all evaluated instances is created

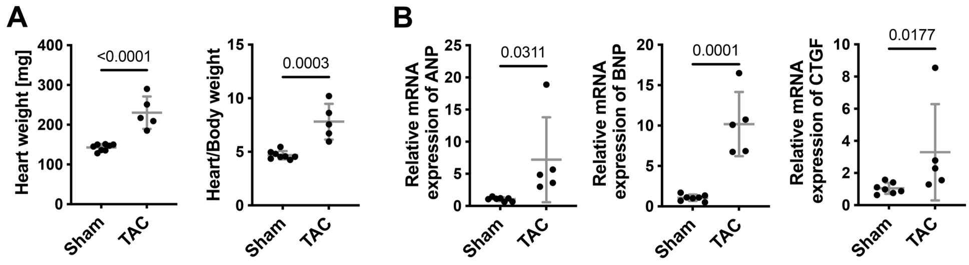

Supplement Fig 3 Basic characterization of TAC-Experiment. **A** Heart weight of sham mice (n = 9) and TAC mice (n = 9) and the heart to body weight ratio. **B** Relative mRNA Expression of ANP, BNP and CTGF in sham and TAC mice

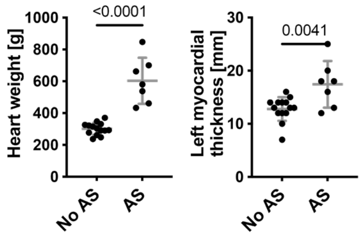

Supplement Fig 4 Heart size of human autopsy cohort. Heart weight and left myocardial thickness of autopsy cases without aortic stenosis (AS) (n = 14) and with AS (n = 7)

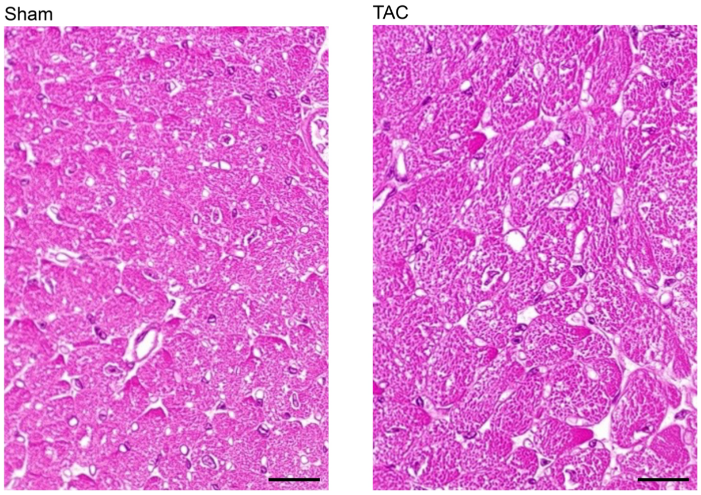

Supplement Fig 5 Representative images of heart cross sections of Sham and TAC, Hematoxylin and eosin staining (scale = 20 µm)

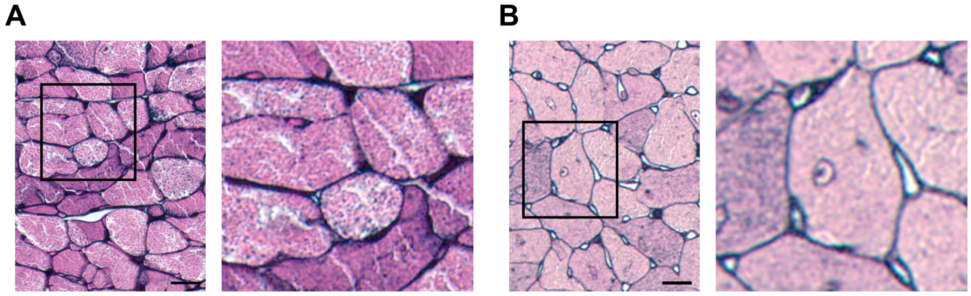

Supplement Fig 6 Challenges in capillary segmentation. **A** Representative cross-section of Gomori silver staining with many not detectable capillaries. **B** Representative cross-section of Gomori silver staining with detectable capillaries (scale = 20 µm)
